# Journal of Negative Results in BioMedicine

**DOI:** 10.1186/1477-5751-13-1

**Published:** 2014-01-20

**Authors:** Daniel Shanahan

**Affiliations:** 1 BioMed Central, 236 Gray's Inn Road, London, WC1X 8HB, UK

## Abstract

**Contributing reviewers:**

A peer-reviewed journal would not survive without the generous time and insightful comments of the reviewers, whose efforts often go unrecognized. Journal of Negative Results in BioMedicine has been blessed by the support of highly-qualified peer reviewers, and the Editor-in-Chief, Bjorn Olsen, and staff of the journal would like to show their appreciation by thanking the following for their invaluable assistance with review of manuscripts for the journal in Volume 12 (2013).

## 

Seena Ajit

United States of America

Mohammed Al-Gayyar

Egypt

Leonard Bielory

United States of America

Leif Bjermer

Sweden

Rollin Brant

Canada

Christine Chambers

Canada

Bradley Chipps

United States of America

Aldo Clerico

Italy

Hans Edmund Eckel

Austria

Michael Eisenhut

United Kingdom

Vera Farah

Brazil

Fran Gengo

United States of America

Barbara Hero

Germany

Melissa Jamerson

United States of America

Timo Koskenkorva

Finland

Shozo Kusachi

Japan

Walter Lehmacher

Germany

Maura Massimino

Italy

Bryony McNeill

Australia

Cathryn Mellersh

United Kingdom

Evelyn Marielle Monninkhof

Netherlands

Takashi Nagasawa

Japan

Anja Niehoff

Germany

Melanie Noel

United States of America

Robert Olney

United States of America

Isabel Palmeirim

Portugal

Michael Polkey

United Kingdom

David Price

United Kingdom

Theodore Price

United States of America

Timothy Prickett

New Zealand

Dieter Reinhardt

Canada

Jan Ringers

Netherlands

Tibor Rohacs

United States of America

Celena Scheede-Bergdahl

Canada

Frode Slinde

Sweden

Roger Stupp

Switzerland

Etsuji Suzuki

Japan

Soshi Takao

Japan

Jean Tchervenkov

Canada

Dag Thelle

Sweden

Samuel Verges

France

Dongfeng Zhang

China

